# Exposure to the Life of a School Child Rather Than Age Determines Myopic Shifts in Refraction in School Children

**DOI:** 10.1167/iovs.63.3.15

**Published:** 2022-03-15

**Authors:** Xiaohu Ding, Ian G. Morgan, Yin Hu, Zhaohui Yuan, Mingguang He

**Affiliations:** 1 State Key Laboratory of Ophthalmology, Zhongshan Ophthalmic Center, Sun Yat-sen University, Guangdong Provincial Key Laboratory of Ophthalmology and Visual Science, Guangdong Provincial Clinical Research Center for Ocular Diseases, Guangzhou, China; 2 Research School of Biology, College of Medicine, Biology and Environment, Australia National University, Canberra, Australia; 3 Centre for Eye Research Australia; Ophthalmology, Department of Surgery, University of Melbourne, Melbourne, Australia

**Keywords:** Myopia, school, education, nearwork, time outdoors

## Abstract

**Purpose:**

The prevalence of myopia increases with both age and grade for children attending school. The current study aimed to distinguish the effects of aging and grade on myopia.

**Methods:**

Grade 1 students (706 at baseline in 2009, mean age 6.56 ± 0.29 years, range 6.00 to 6.99 years old, 55.5% boys) were followed up until 2012. Cycloplegic spherical equivalent (SE) was measured annually.

**Results:**

The sample in a grade was divided into three 4-month age blocks according to their birth month. Within each grade, there were no significant differences in SE between age blocks (all *P* > 0.05), despite an age range of one year. More myopic SE was observed in the youngest block of grade 2 compared to the oldest block of grade 1 (difference, −0.36 ± 0.08 D; *P* < 0.001), although age of the two blocks only differed by four months. Similarly, more myopic SE were found in the youngest block in grade 3 than the oldest block in grade 2 (differences, −0.50 ± 0.10 D; *P* < 0.001) and in the youngest block in grade 4 than the oldest block in grade 3 (differences, −0.82 ± 0.14 D; *P* < 0.001).

**Conclusions:**

Exposure to schooling, rather than age, appears to be the major driver of refractive development, at least in the early years of schooling. Interventions during this period, involving reductions in educational pressure and increased time outdoors may have major effects on the subsequent development of myopia.

It is now generally accepted that the prevalence of myopia has markedly increased over the past few decades, largely driven by environmental changes.^1^^–^^3^ In particular, an unprecedented rise in myopia, up from 10% to 20% of the population to over 80% of the young generation, has been seen in many parts of East Asia.^4^^–^^6^ A similar increase, but of lesser extent, has also been seen in other parts of the world, including the United States and Europe.^7^^,^^8^ Some projections suggest that nearly 50% of the world's population will be myopic by 2050, with around 10% highly myopic.^9^ This is of concern because myopia, particularly high myopia, even when appropriately corrected, is associated with a risk of irreversible blinding complications, such as primary open-angle glaucoma, retinal detachment, and myopic maculopathy.^10^ The increasing prevalence of myopia therefore imposes increased burdens on the health care system and on social support for the visually impaired and blind. In response to this problem, China has now developed a national myopia prevention plan,^11^ and the American Academy of Ophthalmology has proposed developing an integrated program of educational, research, and public health initiatives.^12^

The rapid emergence of a myopia epidemic is not compatible with the slow rate of population genetic changes and must be explained by recent rapid changes in the environment. In general, the increases in myopia began with the introduction and spread of modern education systems and the recent development of systems that aim for universal study with an emphasis on high academic performance.^2^^,^^13^ The latter systems impose heavy loads on students, including high near work activity^14^ and limited time outdoors from the early school years.^15^ Strong evidence for an association between more education and greater development of myopia has been repeatedly reported.^2^^,^^16^ In particular, where children do not go to school or receive limited schooling, there is little development of myopia,^17^^,^^18^ and as school systems have expanded to educate more and more children to a higher level, the prevalence of myopia has increased.^4^^–^^6^^,^^19^^–^^21^ This sort of evidence suggests that the exposures that school-age children experience are likely to be key causal factors in the recent dramatic changes in myopia prevalence. Analysis of the links between years of education and myopia using Mendelian randomization has confirmed a causal link from education to myopia,^22^^,^^23^ as has analysis of the impact of increases in the school leaving age, using Regression Discontinuity analysis.^24^

A confusing factor is that increases in the prevalence of myopia with age are so ubiquitous in modern societies that it is often assumed that children naturally become myopic as they get older. In previous studies, the effect of schooling has almost always been confounded by the synchronous aging of the school children. To resolve the issue, we have used a sample of children who have limited age variation and are educated in the same grade, that can be compared to children of a similar age, who have received more or less education.

In China, there is a strict policy on the age requirements for enrollment in primary school. Children who have turned six years of age by September 1 are qualified to start school, while those who turn six on or after that day will have to start school one year later. That is, children who were born on or before August 31 will be studying in one grade higher than those who were born in the same calendar year but on September 1 or later. Application of this enrollment cutoff defines a group of children within a school grade who differ in age by up to a year, but who receive the same average exposure to schooling and the same deprivation of time outdoors. In the present analysis, we used four-year annual data from a group of Chinese primary school students to estimate the myopic shift induced by every year of life as a schoolchild from the start of Grade 1 to the start of Grade 4.

## Methods

### Participants

Participants in this study were drawn from the control group of the Guangzhou Outdoor Activity Longitudinal (GOAL) Trial, which has been described in detail.^25^ In brief, the GOAL study was a cluster-randomized clinical trial whose purpose was to assess the efficacy of increasing time outdoors in preventing the onset of myopia in Chinese Grade 1 children who were born from September 1, 2002, to August 31, 2003. There were four examinations—on enrollment in Grade 1, with follow-up at the beginning of Grades 2, 3, and 4.

A total of 29 government-operated primary schools in Guangzhou were stratified into six strata based on the prevalence of uncorrected visual acuity by grade in the schools. Two schools in each stratum were randomly selected, one allocated to the intervention group and the other to the control group. Ethics approval was obtained from the human ethics committee of the Zhongshan Ophthalmic Center and the Guangzhou Ministry of Education. This trial was carried out in accordance with the tenets of the Declaration of Helsinki. Written informed consent was obtained from the parents or legal guardians of the children. Only those who gave consent were enrolled at baseline. Additional consent was obtained before the cycloplegic examination at each follow-up visit.

The sample size calculations have been reported for the RCT. In the present analysis, we included all eligible subjects at every visit from the control group. Children whose birth dates were for unknown reasons not compatible with enrollment rules for that grade were excluded. because there appears to be a larger proportion of genetic forms of myopia in children who developed myopia prior to starting school, all subjects who were myopic at the first examination were also excluded.

No participants were involved in setting the research question or the outcome measures, nor were they involved in developing plans for design or implementation of the study. No participants were asked to advise on interpretation or writing up of results.

### Measurement

Eye examinations were performed annually at school, in September and October (the beginning months of each school year), from year 2009 to 2012. The examination team consisted of one senior optometrist, three ophthalmic nurses, and one fellowship-trained ophthalmologist. The examination process began with testing visual acuity at 4 m using a retro-illuminated logarithm of the minimum angle of resolution chart with tumbling-E optotypes (Precision Vision, La Salle, IL, USA). Before cycloplegia, 0.5% proparacaine hydrochloride was used for topical anesthesia. Five minutes later, a single drop of 1% cyclopentolate was administered, then, a second drop of cyclopentolate was administered another five minutes later. A third drop was given after 15 minutes if the pupil diameter remained <6 mm or if the pupillary reflex was still present. This protocol was sufficient to dilate the pupils and eliminate the pupillary response in all participants. Automated refraction (Topcon KR 8900; Topcon Optical Company, Tokyo, Japan) was carried out five times in each eye. Repeated measures were taken until five readings consistent within 0.5D had been obtained. The mean of the five readings was then computed automatically and used in the analyses of the current study. SE was calculated as spherical power plus half cylindrical power. Myopia was defined as SE ≤ −0.50 D.

### Statistics

Analyses were performed using right eye data only, because of the high correlation between right and left eyes at the baseline visit (*r* = 0.924, *P* < 0.001) and at the last visit (*r* = 0.944, *P* < 0.001). According to their birth month, students were divided into three age blocks: Block 1, born from May to August 2003; Block 2, from January to April 2003; and Block 3, from September to December 2002. These covered the youngest through to the oldest children within a grade.

Mean SE and myopia prevalence were calculated and compared in children from the different age blocks educated in the same grade, as well as in different grades. The incidence of myopia was defined as the proportion of participants with myopia who did not have myopia in the previous year or at any earlier visit. One-way analysis of variance, *t*-testing, and χ^2^ testing were adopted where appropriate. Scatter plots and linear best fits of SE by age at examination date were plotted for each age block at the four study visits (from grade 1 to 4).

A *P* value < 0.05 was considered statistically significant. All statistical analyses were performed using Stata 14.0 (Stata Corp, College Station, TX, USA), and the scatter plots with linear line of best fit were created using the g-g plot of R program (https://www.r-project.org).

## Results

**Figure 1.**
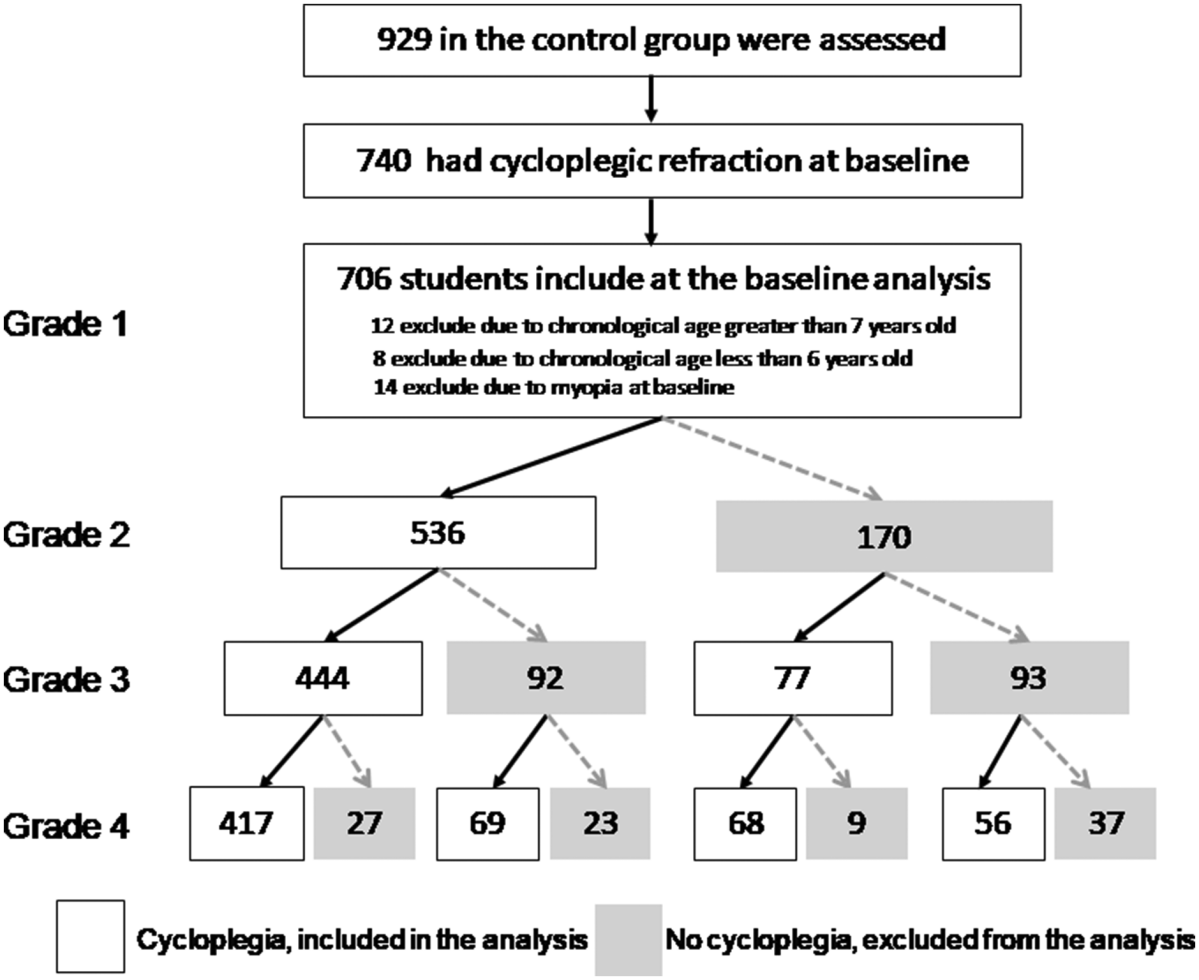
Flowchart of participants in the current analysis from the Guangzhou Outdoor Activity Longitudinal trial.

Figure 1 summarizes the flow of participants included in the current study. At baseline, a total of 929 Grade 1 students were eligible for inclusion in the control group of the GOAL trial. Cycloplegic refractions were successfully performed in 740 children. Among them, 20 children (2.7%) were excluded because of age <6 or ≥7 years, and 14 (2.0%) were excluded because of having myopia at baseline, leaving 706 subjects available for the current analysis. The mean baseline age was 6.56 ± 0.29 years, and 392 (55.5%) were boys. The mean age of the three age blocks at the four study visits is shown in Table 1. The comparison of baseline characteristics between cycloplegia and noncycloplegia students is shown in Supplementary Table S1.

**Figure 2.**
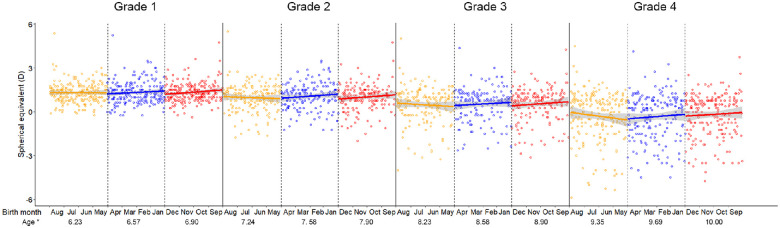
Visualizing the SE change over age blocks. *Average age of each age block. *Colored solid line*: Linear regression line by different age blocks. *Gray line below and above the colored solid line*: The 95% confidence interval for the regression. *Solid vertical line*: The cutoff between different grades. *Dotted vertical line*: The cutoff between different age blocks.

**Table 1.** Age Distributions in Children from Different Age Blocks at the Four Study Visits

	Mean Age		
Visit	Block 1	Block 2	Block 3
Grade 1	6.23 ± 0.10	6.57 ± 0.10	6.90 ± 0.10
Grade 2	7.24 ± 0.10	7.58 ± 0.09	7.90 ± 0.09
Grade 3	8.23 ± 0.10	8.58 ± 0.10	8.90 ± 0.10
Grade 4	9.35 ± 0.10	9.69 ± 0.13	10.00 ± 0.11

Block 1: born May to August 2003; Block 2: born January to April 2003; and Block 3: born September to December 2002.

Mean SE and myopia prevalence in students from the three age blocks at each visit are shown in Table 2. Among three age blocks, mean SE did not differ significantly in children educated in the same grade (one-way analysis of variance, all *P* > 0.05), and prevalence of myopia (χ^2^ test, all *P* > 0.05).

**Table 2.** Spherical Equivalent and Myopia Prevalence in Children From Different Age Blocks at the Four Study Visits

	Spherical Equivalent (D)				Myopia Prevalence (%)			
Visit	Block 1	Block 2	Block 3	*P*	Block 1	Block 2	Block 3	*P*
Grade 1	1.30 ± 0.67	1.31 ± 0.73	1.32 ± 0.64	0.971	—	—	—	
Grade 2	0.96 ± 0.92	1.06 ± 0.88	0.98 ± 0.86	0.558	12/184 (6.52)	11/172 (6.40)	12/180 (6.67)	0.995
Grade 3	0.48 ± 1.20	0.51 ± 1.09	0.52 ± 1.08	0.926	27/178(15.17)	28/163 (17.18)	26/180 (14.44)	0.772
Grade 4	−0.30 ± 1.62	−0.33 ± 1.53	−0.20 ± 1.42	0.663	86/212(40.57)	81/194(41.75)	77/204(37.75)	0.702

Block 1: born May to August 2003; Block 2: born January to April 2003; and Block 3: born September to December 2002.

Within the same grade, comparing block 1 to block 3, the mean age increased about 0.66 years, whereas the SE did not change significantly. However, comparing between grades, even with a lesser age increase of 0.35 years (Block 3 in lower grade vs. Block 1 in higher grade), children who had received one year more of education demonstrated a significantly more myopic SE than those who had received less education (*t*-test, all *P* < 0.01; Table 3). The scatter plots and linear regression lines of SE against age blocks are shown in Figure 2.

**Table 3.** The Comparison of Age and Spherical Equivalent Change Within the Same Grade and Across Grade

	Age Change (y)	*P*	SE Change (D)	*P*
From block 1 to block 3 in the same grade				
Grade 1	0.66 ± 0.01	<0.001	−0.02 ± 0.06	0.802
Grade 2	0.65 ± 0.01	<0.001	−0.02 ± 0.09	0.825
Grade 3	0.66 ± 0.01	<0.001	−0.04 ± 0.12	0.706
From block 3 to block 1 of higher grade				
Grade 1 to grade 2	0.35 ± 0.01	<0.001	−0.36 ± 0.08	<0.001
Grade 2 to grade 3	0.33 ± 0.01	<0.001	−0.50 ± 0.10	<0.001
Grade 3 to grade 4	0.46 ± 0.01	<0.001	−0.82 ± 0.14	<0.001

Block 1: born May to August 2003; Block 2: born January to April 2003; and Block 3: born September to December 2002.

Table 4 shows the changes in SE and myopia incidence in children of three age blocks during the annual periods between two consecutive study visits. With the greatest age variation of eight months (Block 1 vs. Block 3 in the same grade), SE change did not differ in children studying in the same grade (*t*-test, all *P* > 0.05). Nor did incidence of myopia (χ^2^ test, *P* > 0.05). In contrast, with a mean age variation of four months (Block 3 in lower grade vs. Block 1 in higher grade), children in the higher grade had a more negative SE (*t*-test, all *P* < 0.01) and higher myopia incidence (χ^2^ test, all *P* < 0.001), than those in the lower grade.

**Table 4.** Annual Spherical Equivalent Change and Myopia Incidence Change in Children From Each Age Block

	Spherical Equivalent (D)			Myopia Incidence (%)		
Duration	Block 1	Block 2	Block 3	Block 1	Block 2	Block 3
Grade 1 to grade 2	−0.33 ± 0.45	−0.30 ± 0.41	−0.33 ± 0.43	12/184 (6.52)	11/172 (6.40)	12/180 (6.67)
Grade 2 to grade 3	−0.54 ± 0.46	−0.54 ± 0.42	−0.56 ± 0.43	16/151(10.60)	17/141 (12.06)	15/152 (9.87)
Grade 3 to grade 4	−0.65 ± 0.53	−0.67 ± 0.53	−0.68 ± 0.56	30/139 (21.88)	22/133 (16.54)	31/145 (21.38)

Block 1: born May to August 2003; Block 2: born January to April 2003; and Block 3: born September to December 2002.

## Discussion

Using a homogeneous sample with data from repeated annual visits, we found that on average children in the same grade were not significantly different in mean SE despite a maximum age difference of a year, or an average difference of eight months, when comparing birth blocks. There was a slight, statistically insignificant, trend for the oldest children to be less myopic, by 0.1D or less within a grade, compared to the larger, statistically significant changes in mean SE from grade to grade, from around −0.4 D for the oldest children in Grade 1 to the youngest in Grade 2, around −0.5 D from Grade 2 to Grade 3 and around −0.8D from Grade 3 to Grade 4, where the average age difference was only four months. Increases in age associated with the same average educational experience therefore have little impact on SE, whereas marked increases in average educational experience associated with smaller changes in age markedly change SE. Although these results do not establish a causal relationship between the life of a schoolchild and SE, given that a vast body of epidemiological evidence, including evidence from Mendelian randomization^22^^,^^23^ and regression discontinuity^24^ analysis supports a causal link between education and the development of myopia, the most likely explanation of these findings is that exposure to the life of a schoolchild, rather than age, is the major determinant of myopic shifts in refraction, at least in the early primary school years.

Similar conclusions were reached by Xu et al.^26^ from a large study in Wenzhou that measured noncycloplegic refractions. They concluded that the prevalence of myopia increased in a stepwise manner by grade, without being affected by the students’ birth months. However, due to the lack of cycloplegia, the prevalence values reported by Xu et al.^26^ were overestimated. The prevalence of myopia they reported for Grade 1 students was around 10%, consistent with other noncycloplegic estimates.^27^^–^^29^ In contrast, Grade 1 students in the GOALS study showed a prevalence of only 1.4% with cycloplegia, consistent with a number of other reports.^19^^,^^30^^–^^34^

Xu et al.^26^ suggested that the development of myopia should be divided into Stage 1 (Grades 1–6), described as a “myopia-sensitive phase,” and Stage 2 (Grades 7–12), described as a “high myopia–sensitive phase.” Our use of cycloplegic mean SE enabled us to examine refractive development before the onset of myopia, suggesting that their Stage 1 can be usefully subdivided into two stages covering Grades 1–2 and Grades 3–6. The first is particularly interesting because the prevalence of myopia remains low, as it is in preschools. The preschool and early primary school years thus provide an ideal window for the prevention of myopia with increased time outdoors and perhaps with reduced educational pressures before the prevalence of myopia starts to increase. The later increase in the prevalence of high myopia can be simply explained by the fact that onset of myopia in the early primary school years, combined with the high rates of progression typical of East Asia,^35^ leads to the threshold for high myopia first being reached at around the age 11 to 13.^17^^,^^36^ This analysis suggests that delaying the onset of myopia would help to reduce the elevated prevalence of high myopia and could be combined with the effective clinical interventions to control progression that are now available.^37^^–^^43^

In this study, one year of exposure to life as a Grade 1 student resulted in a myopic shift of around −0.3 to −0.4D, whereas one year of exposure to life as a Grade 2 student added around −0.5 D and one year as a Grade 3 student added around −0.8 to mean SE. Because this trial did not beyond Grade 4, we cannot analyze later changes. Changes in refraction analyzed in terms of age have been reported for the Guangzhou Twin Eye Study, which covers children from the age of 7 to 18 to 20,^44^ showing that that annual myopic shifts in refraction reach a peak over ages 9 to 10 (roughly Grade 4) and 10 to 11 (roughly Grade 5) of around −0.4 to −0.7 D/y and then decline markedly during the high school years to almost zero by age 18 to 20.

In the GOAL study, the mean SE of the Grade 1 students at the beginning of the school year was +1.31D. To reach the threshold for myopia (≤−0.5D) thus requires on average a myopic shift in refraction of 1.81D. On average, three years of life as a school child produced a myopic shift in refraction of only 1.58D, resulting in less than half the students becoming myopic by this time. The intervention in the GOAL trial of an additional 40 minutes of time outdoors added to the school day, while maintaining normal classes, produced a reduction of myopic shifts in refraction of 0.15D, leaving the intervention group even further short of the myopia threshold, with a reduction in cases of incident myopia of 23%.^25^ One Mendelian randomization study found a change of −0.17D/y for an additional education with linear regression analysis and −0.27D with Mendelian randomization analysis.^22^ The other gave approximately −0.125D/y with linear regression and −0.46 with Mendelian randomization analysis.^23^ In the raising of the school leaving age study, linear regression analysis gave a change of −0.29D/y, whereas regression discontinuity analysis gave −0.77D.^24^ All these values were obtained on western populations educated in western education systems, and with the exception of the lowest, they are too high to represent an average annual change during 12 years of education, since this would mean that western populations would be substantially myopic. Instead, they probably represent the additional myopia shift associated with an additional year of education at the end of the educational process, which would be quite small, plus the additional myopic shifts through all the previous years of schooling associated with a level of performance that enables an additional year of education to be pursued. Our results show that the myopic shifts in refraction associated with years of schooling vary depending on the level of schooling and accelerate in the Chinese education system through the early primary years before declining later, despite likely increases in educational pressures and greater deprivation of time outdoors because refractive plasticity declines with age.

In this article, we have referred to the experience of a school child rather than to the impact of schooling because our data do not enable us to establish which more specific exposures are involved. Specific risk factor exposures, such as nearwork and limited time outdoors, participation in accelerated educational pathways and in after-school tutorials, have, however, been clearly identified.^16^ However, it is possible that other changes in the life of the schoolchildren also contribute to the differences. Being a schoolchild affects family life, as well as exposures at school.

An important implication of our analysis is that change in mean SE gives us a cumulative measure of the impact of all the risk factors inherent in being a school student, even if we cannot measure the underlying exposures accurately. These exposures are likely to vary between locations, both internationally and within countries, and their differential impact can be seen in the smaller myopic shifts in refraction seen in the Collaborative Longitudinal Evaluation of Ethnicity and Refractive Error Study,^45^ (mean three-year shift in refraction was −0.42D for whites) and in the Sydney Myopia Study,^46^ where the five-year shift in refraction for children of European ancestry was only −0.80D, corresponding to the lower prevalence of myopia generally seen in western populations.^1^^,^^2^^,^^9^

One strategy to reduce the final level of myopia would be to increase the school entry age, while maintaining the school-leaving age, cutting out one year of myopic shifts in refraction. However, such a change might not be compatible with current targets for educational achievement, resulting in compensatory changes in behavioral patterns, either in increased educational pressures, greater deprivation of time outdoors, and greater myopic shifts in refraction within grades, or in the need to add an additional year of schooling at the end of the senior years. These changes would tend to minimize any benefit. An alternative, and perhaps more feasible, strategy would be to change the organization of a school day to minimize educational pressures and maximize opportunities for time outdoors, while maintaining crucial parts of learning in the early school years.

The limitations of this study should be noted. Our sample was limited to Grade 1 to 4. The conclusions cannot be extended to children with older ages or in higher grades, although the results of Xu et al suggest that the conclusions will extend to higher grades.^47^ Another limitation of our study is the relatively small sample size, which means that we have only been able to analyze our results in terms of blocks of birth months, rather than in terms of birth month, but large sample sizes when combined with inferior measurement methodology impose a different set of limitations.

It should be noted that if the data were analyzed at any stage during schooling, apparent effects of month/season of birth on myopia would be seen. However, by the end of schooling, children who started school a year earlier or later will finish schooling a year earlier or later and thus will tend to have had similar average total exposures. This may explain why the evidence for effects of month or season of birth is so contradictory in adults.^47^^,^^48^

These results add further evidence for causal impacts of education on the development of myopia. The minimal average differences in SE that can be seen within grades as compared to the major differences between grades suggest that the major driver of myopic shifts in refraction in school-age children is exposure to the life of a school-age child, with its study pressures and limitations on time outdoors. It is possible that parents also change their attitudes to children as they get older and reach higher grades, and attitudinal changes of this kind may also have a role, although they are unlikely to be as clear-cut as the changes in behavior imposed by school routines. The amount of refractive shift that occurs gives a measure of the total impact of these exposures.

This does not mean that age is completely irrelevant in relation to refractive development. There appears to be a decreasing rate of myopia progression with age in myopes in the later years of schooling,^18^ although educational pressures are more likely to increase—in other words there appears to be an age-dependent decline in overall refractive plasticity. In our data, this would be expected to be manifest in slightly lower changes in the oldest children within a grade. This trend is not obvious in our data but might be detected with larger sample sizes, or in higher grades.

An important implication of these our results is that the efficacy of interventions to reduce educational pressures in the early primary school years can be monitored by looking for reductions in the annual change in refraction, without the need for longer studies to monitor the prevalence of myopia. This should allow simplification of the design of future studies on the efficacy of school-based interventions to reduce the onset and progression of myopia.

## Supplementary Material

Supplement 1
