# Supplementary material for: Exposure to the Life of a School Child Rather Than Age Determines Myopic Shifts in Refraction in School Children
Source: Invest Ophthalmol Vis Sci. 2022 Mar 15;63(3):15. doi: 10.1167/iovs.63.3.15 (PMC8934557; doi:10.1167/iovs.63.3.15)
Supplement: Supplement 1 [file iovs-63-3-15_s001.pdf]

Supplementary

Table S1. The comparison of baseline characteristics between followed students and students lost to follow-up.

	Grade 2			Grade 3			Grade 4		
	Cycloplegia	Non-cycloplegia	P	Cycloplegia	Non-cycloplegia	P	Cycloplegia	Non-cycloplegia	P
N	536	170	--	521	185	--	610	96	--
Baseline age	6.56±0.29	6.56±0.30	0.917	6.56±0.29	6.56±0.29	0.717	6.56±0.29	6.56±0.29	0.886
Baseline SE	1.32±0.68	1.29±0.67	0.562	1.34±0.69	1.22±0.63	0.030	1.31±0.68	1.31±0.66	0.953
Gender (M:F)	287:249	105:65	0.060	281:240	111:74	0.154	344:266	48:48	0.241
